# Bedding out

**Published:** 1991-03

**Authors:** J. L. Burton

**Affiliations:** Consultant Dermatologist, Bristol Royal Infirmary


### Bedding Out

**J.L. Burton**
Consultant Dermatologist, Bristol Royal Infirmary

As I write these words, thick snow blankets my lawn and my *Potentilla* is badly frosted, but you know, we gardeners are never gloomy for long. We know, as we toast our muffins and stir our cocoa, that now is the time to plan next Summer's garden. Of course, with so many gardening programmes on the television nowadays, its quite easy to produce a colourful display. Almost too easy, for the boring fact is that most of your neighbours will have exactly the same 'riot of colour', using the same species from the local Garden Centre. So why not try something different this year, something that will really excite the admiration and curiosity of your friends? Many unusual plants which have never been mentioned on 'Gardeners' World' can easily be cultivated by doctors and I can recommend the following.

For a lovely range of exotic blue and purple bedding plants, you can't beat the *Haematomata*. There are many varieties, ranging in size from the tiny *Purpura* to the giant *Ecchymosis*, but they all share a glowing bluish-purple colour which can last for weeks, before gradually fading to a delicate greenish-yellow. The larger varieties do tend to be tender though, until they're fully established, and may need some protection.

Another of my favourite bedding plants is the *Areola*. Most gardeners are familiar with the lovely pink variety, which admittedly can be quite outstanding, but one of the great attractions for the connoisseur is the wide range of alternative colours. I can't claim to know them all, but I've been most impressed by the coffee-coloured and the dark chocolate variants… I've seen some beauties.

The delicate pink blush of *Virgo intacta* is also well worth searching for, though regrettably it is now becoming something of a rarity. *Virgo* should be disturbed as little as possible and should preferably be left in a bed of its own. Even then it may need protecting with a polythene sheet.

Even more of a rarity nowadays is the *Variola*, though you might still find it stocked by a few specialists. Very similar, though slightly less spectacular, is the *Varicella*, which is still very easy to get, and will produce repeated crops of exhilarating shades of rosy pink.

If you want something a little more florid and long-lasting, why not try the *Rosacea*, which starts with a lovely pink flush and later in the season goes on to a vivid pillar-box red? There are several cultivars but the *Rhinophyma* gives a particularly impressive growth. It takes a while to come to full maturity, but my word, what a show you'll have after a few years. Unfortunately, like many of our more exotic perennials, the mature bloom rarely stays in perfect condition for very long, and at the end of a hot sunny spell, the deep rich red glow is likely to become spoilt by an overgrowth of unsightly yellow spots, which can be difficult to eradicate. If this happens the only real cure is to simply chop off the affected heads and burn them.

All large gardens have their problem areas, of course, where nothing seems to thrive. The first step in overcoming the problem is to determine whether the site is too cold and exposed, too dry or too poorly drained. No matter what the problem though, you will find that there is always something which can cope with that particular environment.

The best thing for a very exposed and windy corner, for instance, might be a hardy dwarf. Some of the more unusual varieties are rather delicate, but *Achondroplasia*, which comes in several colours, is particularly sturdy and will stand a good deal of buffeting. Some types of blue *Cyanosis* will also thrive in cold conditions, but for a particularly cold spot, the purple-blue *Ischaemia* is unbeatable, for the colder it is, the deeper the blue becomes.

If poor drainage is the problem, *Tinea* may be the answer. Several cultivars such as *T. cruris* and *T. pedis* will thrive in extremely moist conditions, especially if the site is fairly warm and sheltered. The lovely little *Legionella* will also stand any amount of water, and the warmer the better.

If your garden has a really dry spot, you'll find *Cystitis* and *Anuria* are ideal. With an established *Cystitis*, lack of water is never a problem, and with *Anuria* in fact the main danger arises from over-watering. Just a few spots each day will be quite enough. Both *Anuria* and the related *Uraemia* also require careful attention to feeding in order to avoid the development of an unhealthy-looking pale yellowish-brown colour at the end of the season. The potassium balance needs particular care.

A common problem in many borders is the development of bare patches in the late Summer. *Alopecia* is a particular culprit in this respect and I try to avoid it for this reason. For really good ground cover there are several creepers like *Impetigo contagiosa* which thrive on neglect and spread like wild-fire. *Impetigo* goes well with the pink *Scabies*, which also spreads readily, often moving from one bed to another in a most mysterious way.

A word of warning about odours might be appropriate here, as several species are unpopular simply because of their heady perfume. *Ketosis* has rather too sickly a scent for many people, and *Halitosis* can be positively overpowering on a warm Summer evening. *Steatorrhoea* too has a strong scent which is not to everyone's liking and one should certainly stay well clear of the indoor cultivar, *Flatus odorata*, which has spoilt many a dinner-party.

Another very stimulating specimen is *Proctalgia fugax*. It is very short-lived, but in a well-sheltered spot it can be exquisite while it lasts. The related *Mastalgia* favours a different site, and will sometimes enjoy considerable exposure. The large overhanging masses may need a lot of support though, if they're to stay at their best throughout the season.

Now a word about a problem which faces most gardeners, the 'eye-sore'. There are very few gardens which do not have some unsightly object like an oil storage-tank or a dilapidated wall, but these can usually be screened by a vigorous and lusty climber such as *Satyriasis*. This will climb up almost anything, and if it has a fault, it is that it tends to propagate too easily. Whatever you do, don't introduce it into a bed of *Virgo intacta*, its much too rampant, though it will often do well in a large bed of *Nymphomania perstans*, when its capable of putting on a spectacular show. *Nymphomania*, by the way, is a very strong and hardy perennial which will stand a good deal of forking, not just in the Autumn, but throughout the year.

Finally let me recommend an exciting specimen for the end of the season, *Arrhythmia*. If that doesn't set your pulse racing, nothing will.

